# Deletion of *Rspo1* or *Rspo3* in the mesenchyme does not affect Wolffian duct maintenance or morphogenesis.

**DOI:** 10.17912/micropub.biology.001942

**Published:** 2026-01-05

**Authors:** Shuai Jia, Jillian Wilbourne, Allyssa Fogarty, Wenyan Bai, Fei Zhao

**Affiliations:** 1 Comparative Biosciences, University of Wisconsin–Madison, Madison, Wisconsin, United States; 2 University of Wisconsin–Madison, Madison, Wisconsin, United States

## Abstract

Secreted proteins, R-Spondin 1 (RSPO1) and R-Spondin 3 (RSPO3), potentiate WNT/β-catenin signaling that play critical roles in reproductive organ development. However, the functional significance of RSPO1 and RSPO3 in Wolffian duct development remains undefined. In this report, we demonstrated their specific expression in the Wolffian duct mesenchyme during sexual differentiation. We generated individual conditional knockouts using *Osr2-Cre* that deleted *Rspo1* or *Rspo3* in the Wolffian duct mesenchyme. Wolffian duct maintenance and morphogenesis was unaffected in either *Rspo1* or *Rspo3* conditional knockout mice. Our results indicate that mesenchymal *Rspo1* or *Rspo3* is dispensable for Wolffian duct development in mice.

**Figure 1. Expression of Rspo1 and Rspo3 in the Wolffian duct mesenchyme and no major effects of conditional Rspo1 or Rspo3 knockout on Wolffian duct development:**
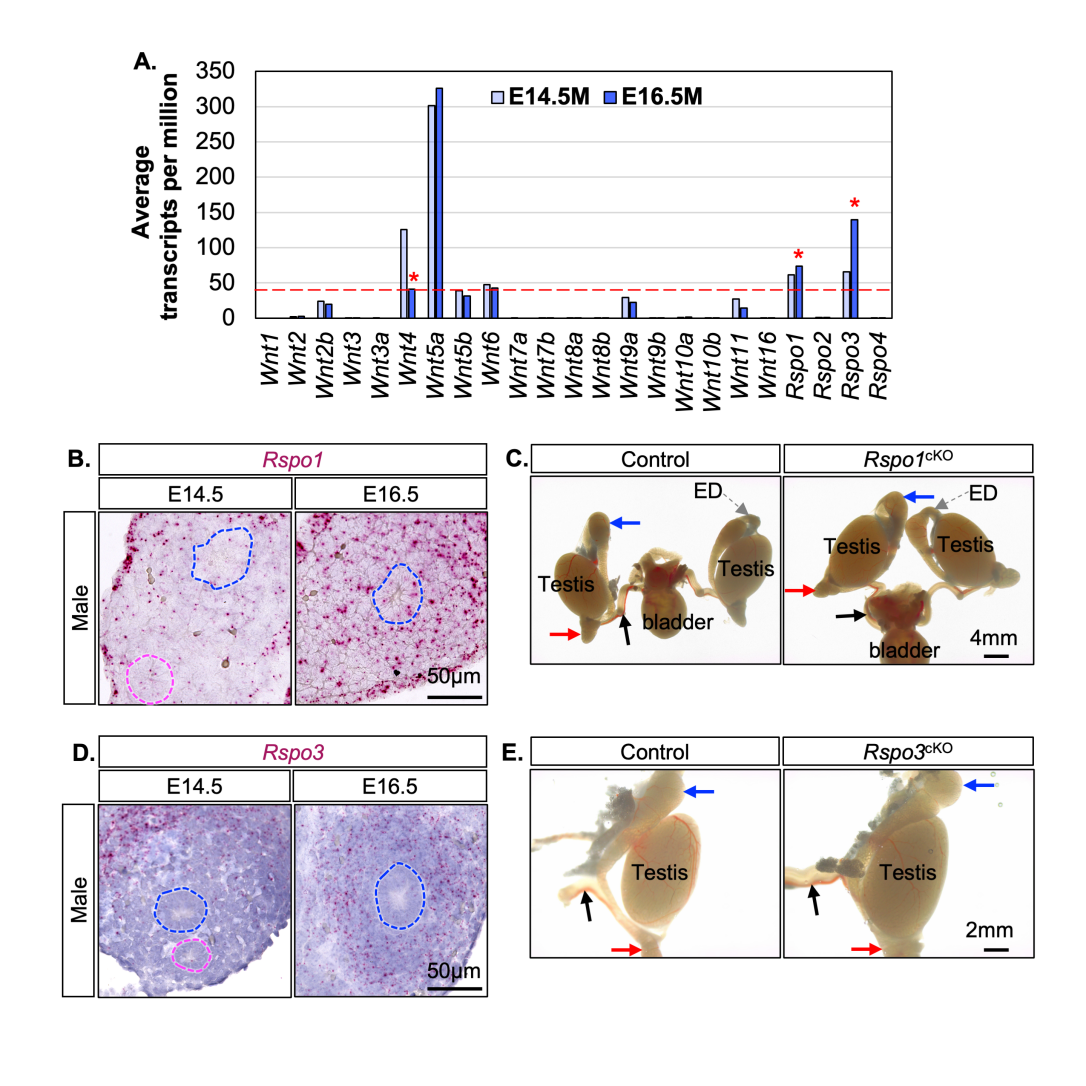
(A) Average expression of 19 *Wnt* genes and 4 *Rspo* genes from our RNA-seq analysis of sorted Wolffian duct mesenchyme of E14.5 and E16.5 male embryos. The entire Wolffian duct including proximal and distal parts were used for sorting. (B) *Rspo1* expression detected by RNAscope in male embryos at E14.5 and E16.5. (C) Bright-field images of testes and epididymides from control and *Rspo1* conditional knockout ( *Rspo1 ^cKO^* ) mice at postnatal day (PND) 21. (D) *Rspo3* expression detected by RNAscope in male embryos at E14.5 and E16.5. (E) Bright-field images of testes and epididymides from control and *Rspo3* conditional knockout ( *Rspo3 ^cKO^* ) mice at PND14. Blue and pink dotted contours in (B & D) represent the Wolffian and Müllerian ducts, respectively. Blue, red, and black arrows in (C & E) indicate cranial epididymides, caudal epididymides, and vas deferens, respectively. The dashed grey arrows in (C) indicate efferent ducts (ED).

## Description

Mesenchymal-epithelial interactions represent a key mechanism in the development of the Wolffian duct, the embryonic precursor of the epididymis, vas deferens and seminal vesicle (Amato et al., 2022; Archambeault et al., 2009; Murashima et al., 2015). Classic tissue recombinant studies demonstrated that paracrine signaling from the mesenchyme governs epithelial morphogenesis and differentiation. For example, when the epithelium from the upper Wolffian duct (future epididymis) was combined with the lower Wolffian duct mesenchyme (future seminal vesicle), the epithelium lost its epididymal identity and adopted seminal vesicle-like structures (Higgins et al., 1989). These mesenchymal effects are in part mediated by secreted mesenchymal factors (Cunha, 2008). Among growth factors, WNTs play a crucial role in organogenesis through stimulating β-catenin (CTNNB1)-dependent and/or -independent intracellular signaling cascades to regulate gene expression and cellular differentiation (Steinhart & Angers, 2018). WNT/β-catenin signaling can be potentiated by R-spondins (RSPOs), which bind to the WNT co-receptors (LGR4-6) and stabilize the WNT receptor complex, thereby enhancing &nbsp;WNT/β-catenin signaling (Steinhart & Angers, 2018). Genetic deletion of either *β-catenin* or *Lgr4* in the Wolffian duct epithelium impairs Wolffian duct morphogenesis (Hoshii et al., 2007; Kumar et al., 2016; Kumar & Tanwar, 2017; Marose et al., 2008; Mendive et al., 2006). These findings suggest that RSPO/WNT signaling may mediate mesenchymal actions in regulating epithelial morphogenesis. However, the identity of mesenchyme-derived WNT ligands and activators that regulate Wolffian duct morphogenesis remains unknown.

To address this knowledge gap, we leveraged our published RNA-seq dataset of sorted *Gli1 ^+^* Wolffian duct mesenchyme from XY embryos on E14.5 and E16.5, corresponding to the onset of Wolffian duct stabilization and coiling (Zhao et al., 2022). We quantified the expression level of all 19 Wnt ligands and 4 *Rspo* family members in these mesenchymal cells using average transcripts per million (TPM, n=3) ( **Fig. 1A** ). *Gli1* expression becomes markedly reduced in the Wolffian duct mesenchyme upon Wolffian duct regression in E16.5 XX embryos (Zhao et al., 2022). Therefore, we used the *Gli1* TPM value in E16.5 XX embryos (TPM=36.5) as a threshold to define minimally expressed genes in the Wolffian duct mesenchyme. Based on this criterion, *Wnt4* , *Wnt5a* , *Wnt6* , *Rspo1* and *Rspo3* exceeded the threshold and emerged as the major expressed WNT factors in the Wolffian duct mesenchyme of XY embryos. Among these candidates, we focus on characterizing expression and function of *Rspo1* and *Rspo3* that were both upregulated at E16.5 compared to E14.5.

We performed RNAscope assays to confirm *Rspo1* expression in the Wolffian duct mesenchyme and its upregulation at E16.5 ( **Fig. 1B** ). *Rspo1* was also expressed in some epithelial cells in male embryos at both E14.5 and E16.5. It is known that *Rspo1* regulates fetal ovarian development (Tomizuka et al., 2008); however, its role in Wolffian duct development remains unclear. To assess the function of mesenchymal *Rspo1* , we used *Osr2-Cre* to generate a mesenchyme-specificconditional knockout *Osr2 ^Cre+^ :Rspo1 ^flox/flox^* ( *Rspo1 ^cKO^* ). In the *Rspo1 ^cKO^* males, the epididymis underwent normal coiling both at birth and puberty ( **Fig. 1C** ), indicating that mesenchymal *Rspo1* is not essential for Wolffian duct maintenance or morphogenesis.

*Rspo3* expression was also significantly increased at E16.5 compared to E14.5 based on our published dataset (Zhao et al., 2022). RNAscope assays confirmed its specific expression in the Wolffian duct mesenchyme and its upregulation at E16.5 in male embryos ( **Fig. 1D** ). RSPO3 has been implicated as an important mesenchyme-derived growth factor driving epithelial differentiation (Dasgupta et al., 2021; Kabiri et al., 2014). To determine its functional significance *,* we used Osr2-Cre to generate a mesenchyme-specificconditional knockout *Osr2 ^Cre+^ :Rspo3 ^flox/flox^* ( *Rspo3 ^cKO^* ). Similar to *Rspo1 ^cKO^* males, *Rspo3 ^cKO^* males exhibited normal epididymal coiling and morphology both at birth and PND14, suggesting that mesenchymal *Rspo3* is not required for Wolffian duct morphogenesis ( **Fig. 1E** ).

Collectively, these results are consistent with the possibility of functional redundancy, which remains to be tested experimentally.

## Methods

**Mice.** *Osr2-Cre (knock-in Cre)* line was provided by Rulang Jiang (Cincinnati Children's Hospital Medical Center) and maintained in its original genetic background (129/Sv X C57BL/6J) (Chen et al., 2009) and was previously established to delete genes in the Wolffian duct mesenchyme prior to E14.5 (Wilbourne et al., 2023). *Rspo3 ^flox/flox^* (Strain #027313) were purchased from The Jackson Laboratory and has been used for conditional gene knockout (Neufeld et al., 2012). *Rspo1 ^flox/flox^* (NM-CKO-2117749, C57BL/6Smoc-Rspo1 ^em1(flox)Smoc^ )was purchased from Shanghai Model Organisms Center, Inc and confirmed by PCR genotyping. Timed mating was set up by housing one stud double heterozygous male with two to three sexually mature flox/flox females in the late afternoon. Vaginal plugs were checked early on the next morning, and the day of plug detection was designated as embryonic day 0.5 (E0.5). The male embryos harboring *Osr2 ^Cre+^ :Rspo1 ^flox/+^ and Osr2 ^Cre+^ :Rspo3 ^flox/+^* were designated as control and those with *Osr2 ^Cre+^ :Rspo1 ^flox/flox^* and *Osr2 ^Cre+^ :Rspo3 ^flox/flox^* as conditional knockout *Rspo1* ^cKO^ and *Rspo3* ^cKO^ , respectively. All procedures involving animals were approved by the University of Wisconsin-Madison (UW-Madison) Animal Care and Use Committees and follow UW-Madison-approved animal study proposals and public laws.

**Genotyping.** PCR was performed with primers &nbsp;(Cre83: 5’-GTCCAATTTACTGACCGTACACC-3’, Cre85: 5’-GTTATTCGGATCATCAGCTACACC-3’) (Chen et al., 2009) for *Osr2-Cre* allele, (Forward: 5’-CCTGGAGTCCAATCCAGAGC-3’,Reverse: 5’-GAGAGCCTTCTGAGCTTGGG-3’) for *Rspo1-flox* allele and (Forward: 5’-TAA TGCCCA GGA ACTTTTGG-3’, Reverse: 5’-GCCTAGAACAGCAACATGGAG-3’) for *Rspo3-flox* allele. Platinum II Taq Hot-Start DNA Polymerase (Invitrogen, 14966001) was used to run the thermal cycle. The conditions for the thermal cycle were 94°C for 2&nbsp;min, 34&nbsp;cycles of [94°C for 15&nbsp;s, 60°C for 5&nbsp;s, and 68°C for 15&nbsp;s] followed by 68°C for 5&nbsp;min.

***Gli1* + mesenchymal cell sorting and RNA-seq.** These experiments were described previously and sequencing results were deposited in the GEO database under the accession code GSE179876 (Zhao et al., 2022). Briefly, Rosa-tdTomato (007909) females were crossed with Gli1-CreER males (007913). Tamoxifen (1 mg/dam) was administered at E12.5 and E13.5. Dams were sacrificed at E14.5 and E16.5; Gli1-CreER⁺; Rosa-tdTomato⁺ mesonephroi were isolated, enzymatically and mechanically dissociated, quenched, pelleted, and resuspended in sorting buffer. tdTomato⁺ cells were sorted on BD FACS Aria II into coated tubes. RNA was extracted (PicoPure kit), and 250 ng was used for library prep (TruSeq RNA non-stranded, Illumina) and sequenced (NextSeq 500, SE 75 bp). Reads were quality-filtered (mean Q < 20), aligned to mm10 (STAR v2.5), counted (featureCounts v1.5.0-p1), and analyzed for differential expression (DESeq2 v1.14.1; FDR < 0.05, |FC| > 1.5, TPM > 1).

**RNAscope** . Tissues were fixed in 10% neutral buffered formalin (Leica, 3800598) overnight at room temperature, underwent three 10-minute 1xPBS washes, and dehydrated by a series of ethanol (70%, 80%, and 95% for 30 min for each; 100% ethanol I and II for 50 min for each). Tissues were then cleared and embedded in paraffin as described (Jia et al., 2022). These embedded tissues were sectioned at 5 μm using a microtome for RNAscope assays following the manufacturer’s protocol as previously described (Zhao et al., 2022). RNAscope Probe- Mm-Rspo3 (ACD, # 402011) and RNAscope Probe- Mm-Rspo1(ACD, # 401991) were used. All sections were then imaged under a compound microscope.
